# Supplementary material for: Protocol for a realist synthesis of health systems responsiveness in low-income and middle-income countries
Source: BMJ Open. 2021 Jun 10;11(6):e046992. doi: 10.1136/bmjopen-2020-046992 (PMC8194331; doi:10.1136/bmjopen-2020-046992)
Supplement: Supplementary data [file bmjopen-2020-046992supp001.pdf]

Literature search for theories of Health Service Responsiveness

Ovid MEDLINE(R) and Epub Ahead of Print, In-Process & Other Non-Indexed Citations and Daily <1946 to November 05, 2020>

Search date: 06-11-2020

Search Strategy:

----

1 conceptual*.tw. (92761)

2 theor*.tw. (656075)

3 ((framework or concep* or logic) adj2 (model* or analy* or evaluat*)).tw. (39784)

4 (reason? adj3 (non-responsiv* or responsiv*)).tw,kw. (42)

5 (factor? adj3 (non-responsiv* or responsiv*)).tw,kw. (4832)

6 (determin* adj3 (non-responsiv* or responsiv*)).tw,kw. (2120)

7 ((concept* or framework or logic or model*) adj3 (non-responsiveness or responsiv*)).tw,kw. (1113)

8 Comment/ (876294)

9 Letter/ (1106450)

10 Editorial/ (545898)

11 "Comment on".ti. (28606)

12 (letter* adj3 editor*).ti. (18269)

13 or/1-12 [Theory Search] (2660960)

14 (health* adj2 system* adj5 respons*).tw,kw. (1718)

15 (health* adj2 delivery* adj5 respons*).tw,kw. (197)

16 (health* adj2 delivery* adj5 (confiden* or trust*)).tw,kw. (78)

17 (health* adj2 system* adj5 (confiden* or trust*)).tw,kw. (640)

18 (health* adj2 system* ad5 satisf*).tw,kw. (0)

19 (health* adj2 delivery* adj5 satisf*).tw,kw. (147)

20 (health* adj2 system* adj5 (prompt or timely or lengthy or delay*)).tw,kw. (540)

21 (health* adj2 delivery* adj5 (prompt or timely or lengthy or delay*)).tw,kw. (61)

22 (health* adj2 system* adj5 dignity).tw,kw. (9)

23 (health* adj2 delivery* adj5 dignity).tw,kw. (0)

24 (health* adj2 system* adj5 (choice? or choose or decide? or decision*)).tw,kw. (1293)

25 (health* adj2 delivery* adj5 (choice? or choose or decide? or decision*)).tw,kw. (348)

26 (health* adj2 system* adj5 access*).tw,kw. (2430)

27 (health* adj2 delivery* adj5 access*).tw,kw. (326)

28 (health* adj2 system* adj5 quality).tw,kw. (2670)

29 (health* adj2 delivery* adj5 quality*).tw,kw. (1240)

30 (health* adj2 system* adj5 accountab*).tw,kw. (252)

31 (health* adj2 delivery* adj5 accountab*).tw,kw. (67)

32 who responsiv*.tw,kw. (11)

33 or/21-32 [Health Service reponsiveness Text word search] (11370)

34 exp *Patient Satisfaction/ (35253)

35 exp *Health Services Accessibility/ (60867)

36 *personal autonomy/ (6110)

37 *respect/ (193)

38 *trust/ (4075)

39 *Confidentiality/ (11479)

40 or/31-39 (116522)

41 exp *"delivery of health care, integrated"/ or *"delivery of health care"/ (69168)

42 40 and 41 [Healthcare Responsivity MeSH search] (3985)

43 33 or 42 [Healthcare Responsivity search] (15163)

52 13 and 43 [Theories of Healthcare Responsivity] (1802)
