# Supplementary material for: Protocol for a realist synthesis of health systems responsiveness in low-income and middle-income countries
Source: BMJ Open. 2021 Jun 10;11(6):e046992. doi: 10.1136/bmjopen-2020-046992 (PMC8194331; doi:10.1136/bmjopen-2020-046992)
Supplement: Author's manuscript [file bmjopen-2020-046992.draft_revisions.pdf]

BMJ Open is committed to open peer review. As part of this commitment we make the peer review history of every article we publish publicly available.

When an article is published we post the peer reviewers' comments and the authors' responses online. We also post the versions of the paper that were used during peer review. These are the versions that the peer review comments apply to.

The versions of the paper that follow are the versions that were submitted during the peer review process. They are not the versions of record or the final published versions. They should not be cited or distributed as the published version of this manuscript.

BMJ Open is an open access journal and the full, final, typeset and author-corrected version of record of the manuscript is available on our site with no access controls, subscription charges or pay-per-view fees (http://bmjopen.bmj.com).

If you have any questions on BMJ Open's open peer review process please email info.bmjopen@bmj.com

BMJ Open

Realist synthesis of key strategies to improve health systems responsiveness to health needs of vulnerable groups in low- and middle-income countries: review protocol

Journal:	*BMJ Open*
Manuscript ID	bmjopen-2020-046992
Article Type:	Protocol
Date Submitted by the Author:	15-Nov-2020
Complete List of Authors:	Mirzoev, Tolib; University of Leeds, Nuffield Centre for International Health and Development<br>Cronin de Chavez, Anna; University of Leeds, Nuffield Centre for International Health and Development<br>Santaella, Ana Manzano; University of Leeds, School of Sociology and Social Policy<br>Kane, Sumit; University of Melbourne Queen's College,<br>Agyepong, Irene; Ghana Health Service<br>Ashinyo , Mary; Ghana Health Service<br>Danso-Appiah, Anthony; University of Ghana, Regional Institute for Population Studies<br>Gyimah, Leveana; Ghana Health Service<br>Yevoo, Lucy; Ghana Health Service<br>Awini, Elizabeth; Ghana Health Service<br>Bui, Ha Thi Thu; Hanoi University of Public Health<br>Do Thi Hanh, Trang; Hanoi University of Public Health<br>Nguyen Thai Quynh , Chi; Hanoi University of Public Health<br>Le, Thi ; Hanoi University of Public Health, Population and Reproductive health<br>Thi Vui, Le; Hanoi University of Public Health<br>Hicks, Joseph; University of Leeds, Nuffield Centre for International Health and Development, Leeds Institute of Health Sciences<br>Wright, Judy; University of Leeds, Leeds Institute of Health Sciences
Keywords:	International health services < HEALTH SERVICES ADMINISTRATION & MANAGEMENT, PUBLIC HEALTH, Organisation of health services < HEALTH SERVICES ADMINISTRATION & MANAGEMENT

SCHOLARONE™
Manuscripts

I, the Submitting Author has the right to grant and does grant on behalf of all authors of the Work (as defined in the below author licence), an exclusive licence and/or a non-exclusive licence for contributions from authors who are: i) UK Crown employees; ii) where BMJ has agreed a CC-BY licence shall apply, and/or iii) in accordance with the terms applicable for US Federal Government officers or employees acting as part of their official duties; on a worldwide, perpetual, irrevocable, royalty-free basis to BMJ Publishing Group Ltd ("BMJ") its licensees and where the relevant Journal is co-owned by BMJ to the co-owners of the Journal, to publish the Work in this journal and any other BMJ products and to exploit all rights, as set out in our licence.

The Submitting Author accepts and understands that any supply made under these terms is made by BMJ to the Submitting Author unless you are acting as an employee on behalf of your employer or a postgraduate student of an affiliated institution which is paying any applicable article publishing charge ("APC") for Open Access articles. Where the Submitting Author wishes to make the Work available on an Open Access basis (and intends to pay the relevant APC), the terms of reuse of such Open Access shall be governed by a Creative Commons licence – details of these licences and which Creative Commons licence will apply to this Work are set out in our licence referred to above.

Other than as permitted in any relevant BMJ Author's Self Archiving Policies, I confirm this Work has not been accepted for publication elsewhere, is not being considered for publication elsewhere and does not duplicate material already published. I confirm all authors consent to publication of this Work and authorise the granting of this licence.

For peer review only

Title: Realist synthesis of key strategies to improve health systems responsiveness to health needs of vulnerable groups in low- and middle-income countries: review protocol.

Authors:

1. Professor Tolib Mirzoev*, University of Leeds, UK t.mirzoev@leeds.ac.uk
2. Dr Anna Cronin de Chavez, University of Leeds, UK A.Chavez@leeds.ac.uk
3. Dr Ana Manzano, University of Leeds, UK A.Manzano@leeds.ac.uk
4. Dr Sumit Kane, University of Melbourne, Australia sumit.kane@unimelb.edu.au
5. Professor Irene Agyepong, Ghana Health Service, Ghana irene.agyepong@gmail.com
6. Dr Mary Ashinyo Ghana Health Service, Ghana keyram1@yahoo.co.uk
7. Dr Anthony Danso-Appiah, University of Ghana, Ghana adanso-appiah@ug.edu.gh
8. Dr Leveana Gyimah, Mental Health Authority, Ghana leveana12@gmail.com
9. Dr Lucy Yevoo, Ghana Health Service lyevoo@gmail.com
10. Dr Elizabeth Awini Ghana Health Service, Ghana awini.elizabeth@gmail.com
11. Prof Bui Thi Thu Ha, Hanoi University of Public Health, Vietnam bth@huph.edu.vn
12. Dr Do Thi Hanh Trang, Hanoi University of Public Health, Vietnam dtht@huph.edu.vn
13. Dr Nguyen Thai Quynh Chi, Hanoi University of Public Health, Vietnam nqc@huph.edu.vn
14. Dr Le Minh Thi, Hanoi University of Public Health, Vietnam lmt@huph.edu.vn
15. Dr Le Thi Vui, Hanoi University of Public Health, Vietnam ltv@huph.edu.vn
16. Dr Joseph Hicks, University of Leeds, UK J.P.Hicks@leeds.ac.uk
17. Mrs Judy Wright, University of Leeds, UK J.M.Wright@leeds.ac.uk

*Corresponding author: Nuffield Centre for International Health and Development, Level 10 Worsley Building, Clarendon Way, Leeds LS2 9NL. t.mirzoev@leeds.ac.uk +44(0)113-343-4869

Keywords:

Responsiveness, health systems, health services, Vietnam, South East Asia, Ghana, sub-Saharan Africa, mental health, realist synthesis, pregnancy

For peer review only

Abstract

Introduction

Health systems responsiveness is a key goal of any health system, yet it is the least studied particularly in low and middle income countries. Research exists on measuring health systems responsiveness highlights seven domains of health systems responsiveness. Little is known, however, about the theoretical underpinning of current understanding of health systems responsiveness, and the mechanisms through which health systems responsiveness works. This review will contribute to bridging this knowledge gap.

Methods and analysis

This review will follow the realist synthesis methodology to investigate health services responsiveness. We will utilise a four-step process, involving: (1) initial screening of literature, (2) formulating initial theories, (3) refining theories and (4) testing and consolidating theories in Ghana and Vietnam. We will include theoretical and conceptual pieces, reviews, empirical studies and grey literature published since 2000. We will explore responsiveness as external and internal interactions within health systems. The search strategy will be purposive and iterative, with screening done throughout. Data extraction will be combined with quality appraisal, using appropriate tools for different study types. Each fragment of evidence will be appraised as it is being extracted, for its relevance to our programme theory of health systems responsiveness. The extracted data pertaining to contexts, mechanisms and outcomes will be synthesised to identify patterns and contradictions. Results will be reported using established guidance on realist syntheses.

Ethics and dissemination

Ethics approvals for the RESPONSE study, which also cover the realist synthesis, were obtained from the University of Leeds School of Medicine Research Ethics Committee (ref: MREC19-051), Ghana Health Service (ref GHS-ERC 012/03/20) and Hanoi University of Public Health (ref 020-149/DD-YTCC).

We will disseminate results of this review through academic papers, conference presentations and stakeholder workshops in Ghana and Vietnam.

Registration details

Prospero registration number: CRD42020200353. Full record is available from: https://www.crd.york.ac.uk/prospero/display_record.php?ID=CRD42020200353

Article summary

Strengths and limitations of this study

For peer review only - http://bmjopen.bmj.com/site/about/guidelines.xhtml

- This paper shares a protocol for a realist synthesis of evidence on key strategies to improve health systems responsiveness to the needs of vulnerable populations in low- and middle-income countries.
- The review will address gaps in the current knowledge on the theoretical underpinnings of health systems responsiveness, and the ways how health systems responsiveness is understood and enacted in the context of low- and middle-income countries.
- The iterative nature of searches, screening and combination of data extraction and quality assessment constitute a strength of realist syntheses, which allows iterative identification of and deeper engagements with, the existing literature, but can be a limitation for example associated with repeated searches and screening at different steps of the review process.
- The large multi-country team will enable drawing on different context-specific understandings of health systems responsiveness, though will also require careful managing and scientific coordination to ensure adequate voice of different perspectives in identifying patterns and contradictions.

For peer review only

For peer review only - http://bmjopen.bmj.com/site/about/guidelines.xhtml

Introduction

Health systems responsiveness is a key goal of any national health system is "…*when institutions… are cognisant and respond appropriately to the universally legitimate expectations of individuals… safeguarding of rights of patients to adequate… care*" [1 p.3]. Improved health systems responsiveness contributes towards equitable improvements in population health. Responsive systems can improve service uptake, ensure adherence to treatment, and ultimately enhance patient welfare[2 3]. People, especially the most vulnerable, are more likely to use services if health systems are responsive to their expectations. Conversely, people are unlikely to use services within unresponsive systems [3-5].

This review is being undertaken as part of the RESPONSE study (https://bit.ly/RESPONSE-project) which seeks to contribute to improving health systems responsiveness in LMICs through case studies of addressing neglected health needs of vulnerable groups in Ghana and Vietnam. It is implemented jointly by the Universities of Leeds, Melbourne, Ghana and Hanoi University of Public Health and the Ghana Health Service.

Rationale

Of all health systems goals which also include improved health, fair financial contribution, efficiency, responsiveness is the least studied particularly in low and middle income countries (LMICs)[6 7]. While research exists on measuring health systems responsiveness using the framework and survey toolkit from the World Health Organisation which highlights seven domains of health systems responsiveness (dignity; autonomy; confidentiality; prompt attention; quality of amenities; access to support networks; and choice of service provider) [1 4 5 8-11], the current literature highlights that interpretations of responsiveness are *context-sensitive* (e.g. expectations of dignity reflect political, democratic and policy climate[4]), and *vary across actors* (e.g. patients and providers with different powers [3 12]) and *health facilities* (e.g. public/private [3 4]). Responsiveness, therefore, is arguably a socially-constructed, rather than an 'absolute' and 'universally normative' concept. Recent work on the topic also emphasises that interactions between the people and their health systems are central to understanding health systems responsiveness[6] and improving responsiveness should therefore address both the 'people' and the 'systems' sides of such interactions[6 13 14].

Little is known, however, about the theoretical underpinning of current understanding of health systems responsiveness and the actual mechanisms through which health systems responsiveness works. This realist synthesis will contribute to bridging this knowledge gap through advancing the theorisation of health system responsiveness and in-depth understanding of key mechanisms of how responsiveness works in specific contexts.

Aim and objectives

In this Realist Synthesis (RS) we will deepen our understanding of theoretical foundations of health systems responsiveness and mechanisms through which health systems responsiveness works (or not) for different health systems actors in Ghana and Vietnam. We will do so through developing and subsequently testing and validating programme theories that will explain which contexts can trigger which mechanisms for health systems responsiveness to produce intended or unintended outcomes. More specifically, this RS will answer the following questions:

For peer review only - http://bmjopen.bmj.com/site/about/guidelines.xhtml

1. Which substantive theories underpin the interpretations of health systems responsiveness, and how they inform these interpretations?
2. What is the understanding of how health systems responsiveness works in different contexts of low and middle-income countries?
3. In what way health system can be responsive to the needs of different health systems actors and specifically the most vulnerable pregnant women in the contexts of LMICs?

The outcome of this review is to develop and subsequently test and refine programme theories of how health systems responsiveness works for different health systems actors, particularly in Ghana and Vietnam.

Methods and analysis

This research employs a realist approach[15 16] to investigate health systems responsiveness. A realist approach will help us make sense of such complexity by identifying how the multiple components interact in non-linear ways. This approach recognises micro, meso and macro contexts (Cs) in triggering the mechanisms (Ms) to produce outcomes (Os) known as CMO configurations to explore what works, in which circumstances, for whom and why [17-22]. Realist synthesis, a form of systematic review, helps to develop in-depth understanding of complex phenomena through articulating programme theories shaped as context-mechanism-outcome (CMO) configurations[15 23]. In this review, while testing, refining and consolidating these programme theories, middle-range theories of how health systems responsiveness interventions work will be proposed.

The basic unit of analysis in realist synthesis (RS) comprises programme theories including their underpinning ideas and assumptions. RS typically involve six inter-related and overlapping steps: identifying review questions, searching for primary studies, quality assessment, data extraction, synthesising results and dissemination[15 23]. In our RS, we will utilise a simplified four-step process[24], which will run alongside other components of the wider RESPONSE study and will be guided by the RAMESES publications standards for realist syntheses[25]:

- Step 1: initial screening to map theoretical landscape of health systems responsiveness and understand key theoretical underpinnings of health systems responsiveness
- Step 2: formulating initial programme theories on the basis of theorisation of health systems responsiveness
- Step 3: refining programme theories through purposeful screening of the literature and iterative consultations with key stakeholders as part of the intervention co-production for improving health systems responsiveness in Ghana and Vietnam
- Step 4: testing programme theories through empirical evaluations of implemented interventions in Ghana and Vietnam and against the substantive theories underpinning health systems responsiveness.

As mentioned earlier, this review is nested within a wider RESPONSE study, which is a mixed-methods Realist Evaluation to be implemented through three broad Phases. In Phase 1, we will understand actors' expectations of responsive health systems, identify key priorities for systems responsiveness, and using evidence from the realist synthesis we will develop an initial programme theory. We will also generate a baseline through reviewing relevant documents and analyse facility records, conduct in-depth interviews, focus groups and community survey. This project Phase will correspond to Steps 1 and 2 in the RS.

For peer review only - http://bmjopen.bmj.com/site/about/guidelines.xhtml

In Phase 2, we will co-produce the context-sensitive interventions to improve health systems responsiveness in Ghana and Vietnam. The interventions will seek to improve internal (i.e. within health system) and external (i.e. people-systems) interactions through participatory workshops with health workers and communities, addressing priorities from Phase 1. The co-production will be through meetings with key local, district, regional and national actors, to be led by relevant health authorities and carefully documented by researchers. This project Phase will correspond to Step 3 in the RS.

In Phase 3, we will implement and evaluate the interventions in Ghana and Vietnam. The implementation will be through existing structures and processes. In the evaluation, we will test and refine our initial theory through comparing the intended design to the interventions' actual performance. We will also assess interventions' feasibility, acceptability and processes. This project Phase will correspond to Step 4 in the RS. In this paper we present the protocol for the planned realist synthesis.

Eligibility

We will include:

1. Theoretical and conceptual pieces, opinions and analyses which focus on responsiveness of health and other public services and systems
2. Reviews: systematic reviews, scoping reviews, meta-syntheses, realist syntheses
3. Empirical studies: including qualitative studies, policy studies, randomised controlled trials (RCTs), quasi-experimental studies and cross sectional and cohort studies;
4. Grey literature, including discussion pieces, reports, policies, plans and guidelines in Ghana and Vietnam and relevant local-level documentation within health facilities.

We will exclude:

1. Empirical studies published before 2000, i.e. when the WHO introduced the current concept of health systems responsiveness
2. Studies in languages where we are unable to resource translation
3. Studies with no full text available for subsequent analysis

No restrictions on settings or other characteristics for the initial searches and understanding the theoretical foundations of health systems responsiveness. The context for empirical literature on health systems responsiveness will be restricted to LMICs only.

The review is being conducted by a team comprising speakers of English, Spanish, French, Russian and Vietnamese languages. While initial screening will be conducted in either of these languages, analysis will be conducted in English so we will either aim to obtain an English copies of papers or arrange for a translation.

Participants

Health systems responsiveness affects a range of different populations such as communities, individuals (particularly pregnant women), service providers, facility managers, regional/province and national-level policymakers. There will be no restrictions on populations in our review, and papers will be included if they (1) contribute to building the theory of how responsiveness is supposed to work or (2) contribute to testing those theories particularly in Ghana and Vietnam.

For peer review only - http://bmjopen.bmj.com/site/about/guidelines.xhtml

For peer review only

Interventions

Health systems responsiveness is reflected in two types of interactions: internal interactions (within health systems e.g. between service providers and managers) and external interactions (service users and health systems)[6].

Comparison / control

Not applicable to this review.

Outcome

We will focus on health systems responsiveness to health needs of local populations, particularly vulnerable groups. As well as seeking to understand the general concept of responsiveness of health systems in LMICs we will consider the seven domains under the WHO framework for health systems responsiveness: dignity; autonomy; confidentiality; prompt attention; quality of amenities; access to support networks; and choice of service provider[1].

We will particularly explore the interactions between the people and their health systems as being central to the current understanding of responsiveness[6] and improving responsiveness should therefore address both the 'people' and the 'systems' sides of such interactions[6 13 14]. Evidence also shows that interpretations of responsiveness can be context-sensitive (e.g. expectations of dignity reflect political, democratic and policy climate[4]), and vary across actors (e.g. patients and providers with different powers[3 12]) and health facilities (e.g. public/private[3 4]). Therefore, we will study responsiveness as a socially-constructed, rather than an 'absolute' and normative concept.

The overall longer-term outcome to be studied in this review is improved health systems responsiveness to the needs of different health systems actors, reflected in improvements in two types of interactions: internal interactions (within health systems e.g. between service providers and managers) and external interactions (service users and health systems)[6]. We will also aim to understand changes in the individual elements of responsiveness (e.g. dignity, confidentiality) as distinct outcomes, which will be refined as our programme theories will develop over the course of the review.

Study records

Searches

Throughout all steps, the search strategy will be purposive, and as such will evolve as our programme theories develop over time. In answering the first review question on theorisation of health systems responsiveness (during steps 1 and 2), our searches will not be limited to any geographic area, whereas our search for empirical literature on health systems responsiveness in

For peer review only - http://bmjopen.bmj.com/site/about/guidelines.xhtml

answering review questions 2 and 3 (during steps 3 and 4) will specifically focus on low- and middle-income countries and where appropriate specifically on Ghana and Vietnam.

At step 1 and 2, we will search Medline (1946+) and Google Scholar for reports containing substantive theories and frameworks. MeSHs, free text words and synonyms will be used for the following search concepts (blocks):

- health systems responsiveness: responsiveness/satisfaction/confidence/trust and WHO elements of responsiveness: dignity; autonomy; confidentiality; prompt attention; quality of amenities; access to support networks; and choice of service provider
- theories: theory; framework; conceptualisation

At step 3 and 4, the searches for empirical literature on health systems responsiveness in LMICs will be determined by the choice of initial programme theory(s) at the end of step 2. We anticipate the search question will be to identify evidence that confirms or refutes the theory in different contexts:

- different health systems actors: communities/pregnant women/service providers/doctors/nurses/health workers/ health managers/health providers/policymakers/decision-makers/implementers/researchers/
- LMIC regional contexts, specifically sub-Saharan Africa and South East Asia (including lists of relevant countries)

Our searches will include MeSHs, free text words and synonyms for each of search concepts in addition to the theory or mechanism of action under investigation. It is likely we will search:

1. Published literature from some of the following scientific databases: Applied Social Science Index Abstracts, CINAHL, Global Health, Maternity and Infant Care Database, Medline, PsycINFO, Scopus, Web of Science.
2. Grey literature from main gateways (e.g. ELDIS) and relevant organisations (e.g. World Health Organisation repository; and MeasureEvaluation).
3. Policy and regulatory documentation from Ghana and Vietnam.

A sample Medline search strategy for step 1 and 2 is available in a supplementary file.

Searching for both theory and evidence for realist reviews can be highly iterative in nature. Multiple searches will be conducted using a variety of search techniques as appropriate[26]. Conventional database searches will be complemented with CLUSTER searches techniques such as citation snowballing and project searching (e.g. WHO responsiveness tool project)[27]. These techniques will be used to identify related relevant studies and 'contextually rich' evidence.

Data management

Search results will be managed using EndNote version X9 software. Records will be uploaded into Rayyan software (https://rayyan.qcri.org/welcome) for screening.

Qualitative data analysis software NVIVO will be used for organising and managing data, cross referencing concepts, actors and populations. Data extraction will be done with Microsoft Word or Excel.

For peer review only - http://bmjopen.bmj.com/site/about/guidelines.xhtml

Screening

Screening in RS is typically done throughout the different stages of the review. In our case, we will conduct:

- initial screening for theoretical frameworks and models of health systems responsiveness during the first step to inform development of programme theories, and then
- purposeful screening of the literature for refining our programme theories of how health systems responsiveness works, alongside the consultations with key stakeholders as part of the intervention co-production for improving health systems responsiveness in Ghana and Vietnam

Initial and subsequent screening will be done collaboratively by the different members of the review team, and a minimum of 20% of results will be double-screened for calibration and quality control.

Data extraction

The realist syntheses have no standardised common data extraction form and data extraction is often combined with quality appraisal of studies[15 23 24]. As RS involves a highly iterative process where search terms and concepts will be continually refined and developed throughout, the process will be documented carefully.

At each step a data extraction table in Microsoft Word or Excel format, will be used to track included papers adapting the following headings which will enable us to advance our programme theories:

- key paper identifiers (full citation)
- type of paper (published, grey literature) and its source
- elements of health systems responsiveness addressed as per established frameworks for health systems responsiveness[1 6]
- links to our programme theory(ies) showing causality of key contexts triggering specific mechanisms to produce outcomes

Retroductive analysis and coding for each stage of the review will be used. An international team of reviewers will also seek input from stakeholders through focus groups and a community survey in Ghana and Vietnam to inform the construction of the Context-Mechanism-Outcome configurations.

Quality assessment and risk of bias

Quality appraisal, data extraction and stakeholder opinions are typically combined in RS, the process which we will also follow in this review. All studies will be considered for their ability to provide data related to context, mechanisms and outcomes. Quality appraisal in RS is usually done on case-by-case basis and we will adapt and utilise appropriate tools (e.g. JBI or CASP tools) and relevant guidance (e.g. RAMESES standards).

Assessing relevance and study quality will be undertaken collaboratively through regular team discussions. Each fragment of evidence will be appraised as it is being extracted, for its relevance to our programme theory of health systems responsiveness and the methodological rigour with which it has been produced, rather than the study as a whole.

For peer review only - http://bmjopen.bmj.com/site/about/guidelines.xhtml

Data synthesis and analysis

Analysis of sub-groups is an important feature of RS, though the sub-group analysis normally emerges as part of the theory elicitation process. Therefore, it would be inappropriate to narrowly pre-determine the subgroups.

In the first step of the review, where we will be establishing theories, we will not be restricting the review to any geographical, health condition or population sub-sets. As the review develops, we may focus searches for empirical literature on health service responsiveness in LMICs (particularly Vietnam/South East Asia and Ghana/sub-Saharan Africa). We are also interested in maternal mental health as a condition sub-set, and pregnant women as well as other health systems actors such as service providers and managers as a population sub-set. However, the subgroup analysis will be driven by the theory elicitation process conducted during the first two steps of the review.

We will relate results of our analyses within these subsets to the individual elements of health systems responsiveness and systems' responsiveness as a whole. All these will allow us to apply the findings of the review in a practical way through the co-design of interventions for these conditions for these populations.

Strategy for data synthesis

The extracted data pertaining to contexts, mechanisms and outcomes will be synthesised to identify patterns and contradictions in the relationships between contexts, mechanisms and outcomes. These relationships will be first documented and will inform initial theory, and subsequently will be compared to the initial theory to inform theory refinement and development. Data synthesis will also search for, develop and/or refine substantive theory to further explain the substantive theory. At this stage any middle range theories will also be identified and extracted.

Our synthesis will be specifically concerned with understanding the conditions under which health systems responsiveness works (or not) for different health systems actors in Ghana and Vietnam. Data synthesis in RS takes several forms. Realist synthesis entails a form of 'triangulation', bringing together information from different studies to explain why a pattern of outcomes may occur. Our review would go further and would identify explanations for potentially contrasting (or similar) findings to identify the circumstances in which the intended mechanism and intended outcomes occur (improved health systems responsiveness) and those in which unintended mechanisms and outcomes (sense of mutual distrust or lack of confidence) occur. Thus, it seeks to provide an explanation for the whole pattern of outcomes across studies rather than seek out an average effect.

We will specifically synthesise data along each element of health systems responsiveness (the internal and external interactions[6] and seven elements of responsiveness[1]). These will relate closely to our programme theory and further elements of data synthesis may be added to capture the causality and contingent nature of the contexts, mechanisms and outcomes of health systems responsiveness. Reporting of results of this realist synthesis will follow established guidance on realist syntheses[15 23]

For peer review only - http://bmjopen.bmj.com/site/about/guidelines.xhtml

Patient and public involvement

This review will use the previously published data. Therefore, no patient and public involvement in during step 1 (theorisation of health systems responsiveness). However, patient and public involvement will be included in the planned engagements with key stakeholders within step 2 (formulating initial programme theory of health systems responsiveness through initial consultations), step 3 (refining programme theories through co-production of interventions for improving health systems responsiveness) and step 4 (testing programme theories through empirical evaluations of implemented interventions).

The nature of patient and public involvement in the review would be reflective of the nature of the review steps. During step 2, their involvement would be as sources of local knowledge in identifying priority areas for health systems responsiveness. During step 3, their involvement would comprise more active participation in the co-design of the interventions, and during step 4 patient and public involvement would become more evaluative of the changes achieved by the implemented interventions on degree of health systems responsiveness to their needs.

Ethics and dissemination

Ethics approvals for the wider RESPONSE study, which also cover the primary data collection for the realist synthesis, were obtained from the University of Leeds School of Medicine Research Ethics Committee (ref: MREC19-051), Ghana Health Service (ref GHS-ERC 012/03/20) and Hanoi University of Public Health (ref 020-149/DD-YTCC).

We will disseminate results through academic papers and stakeholder workshops in Ghana and Vietnam, during which review results will also be complemented by results of reviews of policy documents. The findings of the review will also be presented at scientific conferences such as the Global Symposia on Health Systems Research and Thematic Working Groups of the Health Systems Global.

References

1. de Silva A. A framework for measuring responsiveness. GPE Discussion Paper series No 32. Geneva: World Health Organization, 2000.
2. Lodenstein E, Dieleman M, Gerretsen B, et al. A realist synthesis of the effect of social accountability interventions on health service providers' and policymakers' responsiveness. *Systematic Reviews* 2013;2(1):98.
3. Valentine N, Prasad A, Rice N, et al. Health Systems Responsiveness – A Measure of the Acceptability of Health Care Processes and Systems. In: Smith P, Mossialos E, S L, eds. Performance Measurement for Health System Improvement: Experiences, Challenges and Prospects. London: WHO European Regional Office 2009:pp. 138–86.

For peer review only - http://bmjopen.bmj.com/site/about/guidelines.xhtml

4. Robone S, Rice N, Smith PC. Health Systems' Responsiveness and Its Characteristics: A Cross-Country Comparative Analysis. *Health Serv Res* 2011;46(6pt2):2079-100. doi: 10.1111/j.1475-6773.2011.01291.x
5. Darby C, Valentine N, Murray CJL, et al. World Health Organization: Strategy on measuring responsiveness. *The Journal of Medicine and Philosophy* 2000;39(1):2.
6. Mirzoev T, Kane S. What is health systems responsiveness? Review of existing knowledge and proposed conceptual framework. *BMJ Global Health* 2017;2(4):e000486. doi: 10.1136/bmjgh-2017-000486
7. Lodenstein E, Dieleman M, Gerretsen B, et al. Health provider responsiveness to social accountability initiatives in low- and middle-income countries: a realist review. *Health Policy Plan* 2016;32(1):125-40. doi: 10.1093/heapol/czw089
8. Letkovicova H, Prasad A, Vallée RL, et al. The health systems responsiveness analytical guidelines for surveys in the multi-country survey study. Geneva: World Health Organization 2005.
9. Chao J, Lu B, Zhang H, et al. Healthcare system responsiveness in Jiangsu Province, China. *BMC Health Services Research* 2017;17(1):31. doi: 10.1186/s12913-017-1980-2
10. Forouzan S, Padyab M, Rafiey H, et al. Measuring the Mental Health-Care System Responsiveness: Results of an Outpatient Survey in Tehran. *Frontiers in Public Health* 2015;3:285. doi: 10.3389/fpubh.2015.00285
11. Malhotra C, Do YK. Public health expenditure and health system responsiveness for low-income individuals: results from 63 countries. *Health Policy Plan* 2017;32(3):314-19. doi: 10.1093/heapol/czw127 [published Online First: 2016/09/22]
12. Valentine NB, Bonsel GJ. Exploring models for the roles of health systems' responsiveness and social determinants in explaining universal health coverage and health outcomes. *Global Health Action* 2016;9:10.3402/gha.v9.29329. doi: 10.3402/gha.v9.29329
13. Cleary S, Molyneux S, Gilson L. Resources, attitudes and culture: an understanding of the factors that influence the functioning of accountability mechanisms in primary health care settings. *BMC Health Services Research* 2013;13(1):320.
14. Berlan D, Shiffman J. Holding health providers in developing countries accountable to consumers: a synthesis of relevant scholarship. *Health Policy Plan* 2012;27(4):271-80. doi: 10.1093/heapol/czr036
15. Pawson R, Greenhalgh T, Harvey G, et al. Realist synthesis: an introduction. ESRC Research Methods Programme, Working Paper Series. 2004.
16. Pawson R, Tilley N. Realistic Evaluation: London: SAGE Publications 1997.
17. Greenhalgh T, Pawson R, Wong G, et al. 'Theory' in realist evaluation: The Rameses II project 2017.
18. Greenhalgh T, Pawson R, Wong G, et al. What is a mechanism? What is a programme mechanism?: The Rameses II project 2017.
19. Greenhalgh T, Pawson R, Wong G, et al. What realists mean by context; or, Why nothing works everywhere or for everyone: The Rameses II project 2017.
20. Manzano A. The craft of interviewing in realist evaluation. *Evaluation* 2016;22(3):342–60. doi: 10.1177_1356389016638615
21. Mirzoev T, Etiaba E, Ebenso B, et al. Study protocol: realist evaluation of effectiveness and sustainability of a community health workers programme in improving maternal and child health in Nigeria. *Implementation Science* 2016;11(1):1-11. doi: 10.1186/s13012-016-0443-1
22. Pawson R, Manzano-Santaella A. A realist diagnostic workshop. *Evaluation* 2012;18(2):176-91. doi: 10.1177/1356389012440912
23. Rycroft-Malone J, McCormack B, Hutchinson AM, et al. Realist synthesis: illustrating the method for implementation research. *Implementation Science* 2012;7(1):33. doi: 10.1186/1748-5908-7-33

For peer review only - http://bmjopen.bmj.com/site/about/guidelines.xhtml

24. Cooper C, Lhussier M, Shucksmith J, et al. Protocol for a realist review of complex interventions to prevent adolescents from engaging in multiple risk behaviours. *BMJ Open* 2017;7(9):e015477. doi: 10.1136/bmjopen-2016-015477
25. Wong G, Greenhalgh T, Westhorp G, et al. RAMESES publication standards: realist syntheses. *BMC Medicine* 2013;11(1):21. doi: 10.1186/1741-7015-11-21
26. Booth A, Briscoe S, Wright JM. The "realist search": A systematic scoping review of current practice and reporting. *Research Synthesis Methods* 2020;11(1):14-35. doi: https://doi.org/10.1002/jrsm.1386
27. Booth A, Harris J, Croot E, et al. Towards a methodology for cluster searching to provide conceptual and contextual "richness" for systematic reviews of complex interventions: case study (CLUSTER). *BMC Medical Research Methodology* 2013;13(1):118. doi: 10.1186/1471-2288-13-118

Authors' contributions

TM and SK conceived the study; All authors jointly wrote the protocol and read and approved the final version of the manuscript.

Funding statement

The research reported in this paper received funding from the Joint MRC/ESRC/DFID/Wellcome Health Systems Research Initiative (grant ref: MR/T023481/1). The views expressed in this publication are those of the author(s) and not necessarily those of the funders.

Competing interests statement

None declared

Word count

3,222 words

For peer review only - http://bmjopen.bmj.com/site/about/guidelines.xhtml

Manuscript Title**:** Realist synthesis of key strategies to improve health systems responsiveness to health needs of vulnerable groups in low- and middle-income countries: review protocol.

Checklist for reporting realist evaluations. Source: Wong G, Greenhalgh T, Westhorp G, et al. RAMESES publication standards: realist syntheses. *BMC Medicine* 2013;11(1):21. doi: 10.1186/1741-7015-11-21 https://bmcmedicine.biomedcentral.com/articles/10.1186/1741-7015-11-21

**TITLE**			**Included Y/N**	**Page No**
1		In the title, identify the document as a realist synthesis or review	Y	1
ABSTRACT				
2		While acknowledging publication requirements and house style, abstracts should ideally contain brief details of: the study's background, review question or objectives; search strategy; methods of selection, appraisal, analysis and synthesis of sources; main results; and implications for practice.	Y	2
INTRODUCTION				
3	Rationale for review	Explain why the review is needed and what it is likely to contribute to existing understanding of the topic area.	Y	4
4	Objectives and focus of review	State the objective(s) of the review and/or the review question(s). Define and provide a rationale for the focus of the review.	Y	4-5
METHODS				
5	Changes in the review process	Any changes made to the review process that was initially planned should be briefly described and justified.	n/a for protocols	n/a
6	Rationale for using realist synthesis	Explain why realist synthesis was considered the most appropriate method to use.	Y	4
7	Scoping the literature	Describe and justify the initial process of exploratory scoping of the literature.	Y	7-8
8	Searching processes	While considering specific requirements of the journal or other publication outlet, state and provide a rationale for how the iterative searching was done. Provide details on all the sources accessed for information in the review. Where searching in electronic databases has taken place,	Y	7-8

For peer review only

TITLE			Included Y/N	Page No
		the details should include, for example, name of database, search terms, dates of coverage and date last searched. If individuals familiar with the relevant literature and/or topic area were contacted, indicate how they were identified and selected.		
9	Selection and appraisal of documents	Explain how judgements were made about including and excluding data from documents, and justify these.	Y	6
10	Data extraction	Describe and explain which data or information were extracted from the included documents and justify this selection.	Y	9
11	Analysis and synthesis processes	Describe the analysis and synthesis processes in detail. This section should include information on the constructs analyzed and describe the analytic process.	Y	10
RESULTS				
12	Document flow diagram	Provide details on the number of documents assessed for eligibility and included in the review with reasons for exclusion at each stage as well as an indication of their source of origin (for example, from searching databases, reference lists and so on). You may consider using the example templates (which are likely to need modification to suit the data) that are provided.	n/a for protocols	n/a
13	Document characteristics	Provide information on the characteristics of the documents included in the review.	n/a for protocols	n/a
14	Main findings	Present the key findings with a specific focus on theory building and testing.	n/a for protocols	n/a
DISCUSSION				
15	Summary of findings	Summarize the main findings, taking into account the review's objective(s), research question(s), focus and intended audience(s).	n/a for protocols	n/a
16	Strengths, limitations and future research directions	Discuss both the strengths of the review and its limitations. These should include (but need not be restricted to) (a) consideration of all the steps in the review process and (b) comment on the overall strength of evidence supporting the explanatory insights which emerged.<br>The limitations identified may point to areas where further work is needed.	Y	1-2

For peer review only

TITLE			Included Y/N	Page No
17	Comparison with existing literature	Where applicable, compare and contrast the review's findings with the existing literature (for example, other reviews) on the same topic.	n/a for protocols	n/a
18	Conclusion and recommendations	List the main implications of the findings and place these in the context of other relevant literature. If appropriate, offer recommendations for policy and practice.	n/a for protocols	n/a
19	Funding	Provide details of funding source (if any) for the review, the role played by the funder (if any) and any conflicts of interests of the reviewers.	Y	13

For peer review only

BMJ Open

Protocol for a realist synthesis of health systems responsiveness in low- and middle-income countries

Journal:	*BMJ Open*
Manuscript ID	bmjopen-2020-046992.R1
Article Type:	Protocol
Date Submitted by the Author:	13-May-2021
Complete List of Authors:	Mirzoev, Tolib; London School of Hygiene & Tropical Medicine, Global Health and Development<br>Cronin de Chavez, Anna; London School of Hygiene & Tropical Medicine, Global Health and Development<br>Manzano, Ana; University of Leeds, School of Sociology and Social Policy<br>Agyepong, Irene; Ghana Health Service<br>Ashinyo, Mary Eyram; Ghana Health Service<br>Danso-Appiah, Anthony; University of Ghana, Regional Institute for Population Studies<br>Gyimah, Leveana; Ghana Health Service<br>Yevoo, Lucy; Ghana Health Service<br>Awini, Elizabeth; Ghana Health Service<br>Bui, Ha Thi Thu; Hanoi University of Public Health<br>Do Thi Hanh, Trang; Hanoi University of Public Health<br>Nguyen, Quynh-Chi; Hanoi University of Public Health<br>Le, Thi ; Hanoi University of Public Health, Population and Reproductive health<br>Thi Le, Vui; Hanoi University of Public Health<br>Hicks, Joseph; University of Leeds, Nuffield Centre for International Health and Development, Leeds Institute of Health Sciences<br>Wright, Judy; University of Leeds, Leeds Institute of Health Sciences<br>Kane, Sumit; University of Melbourne Queen's College,
**Primary Subject Heading**:	Global health
Secondary Subject Heading:	Global health, Health policy, Health services research
Keywords:	International health services < HEALTH SERVICES ADMINISTRATION & MANAGEMENT, PUBLIC HEALTH, Organisation of health services < HEALTH SERVICES ADMINISTRATION & MANAGEMENT

SCHOLARONE™
Manuscripts

I, the Submitting Author has the right to grant and does grant on behalf of all authors of the Work (as defined in the below author licence), an exclusive licence and/or a non-exclusive licence for contributions from authors who are: i) UK Crown employees; ii) where BMJ has agreed a CC-BY licence shall apply, and/or iii) in accordance with the terms applicable for US Federal Government officers or employees acting as part of their official duties; on a worldwide, perpetual, irrevocable, royalty-free basis to BMJ Publishing Group Ltd ("BMJ") its licensees and where the relevant Journal is co-owned by BMJ to the co-owners of the Journal, to publish the Work in this journal and any other BMJ products and to exploit all rights, as set out in our licence.

The Submitting Author accepts and understands that any supply made under these terms is made by BMJ to the Submitting Author unless you are acting as an employee on behalf of your employer or a postgraduate student of an affiliated institution which is paying any applicable article publishing charge ("APC") for Open Access articles. Where the Submitting Author wishes to make the Work available on an Open Access basis (and intends to pay the relevant APC), the terms of reuse of such Open Access shall be governed by a Creative Commons licence – details of these licences and which Creative Commons licence will apply to this Work are set out in our licence referred to above.

Other than as permitted in any relevant BMJ Author's Self Archiving Policies, I confirm this Work has not been accepted for publication elsewhere, is not being considered for publication elsewhere and does not duplicate material already published. I confirm all authors consent to publication of this Work and authorise the granting of this licence.

For peer review only

Title: Protocol for a realist synthesis of health systems responsiveness in low- and middle-income countries.

Authors:

1. Prof Tolib Mirzoev*, London School of Hygiene and Tropical Medicine, UK tolib.mirzoev@lshtm.ac.uk
2. Dr Anna Cronin de Chavez, London School of Hygiene and Tropical Medicine, UK Anna.Chavez@lshtm.ac.uk
3. Dr Ana Manzano, University of Leeds, UK A.Manzano@leeds.ac.uk
4. Prof Irene Akua Agyepong, Ghana Health Service, Ghana irene.agyepong@gmail.com
5. Dr Mary Eyram Ashinyo Ghana Health Service, Ghana keyram1@yahoo.co.uk
6. Dr Anthony Danso-Appiah, University of Ghana, Ghana adanso-appiah@ug.edu.gh
7. Dr Leveana Gyimah, Mental Health Authority, Ghana leveana12@gmail.com
8. Dr Lucy Yevoo, Ghana Health Service lyevoo@gmail.com
9. Dr Elizabeth Awini Ghana Health Service, Ghana awini.elizabeth@gmail.com
10. Prof Ha Thi Thu Bui, Hanoi University of Public Health, Vietnam bth@huph.edu.vn
11. Dr Trang Thi Hanh Do, Hanoi University of Public Health, Vietnam dtht@huph.edu.vn
12. Dr Quynh-Chi Thai Nguyen, Hanoi University of Public Health, Vietnam nqc@huph.edu.vn
13. Dr Thi Minh Le, Hanoi University of Public Health, Vietnam lmt@huph.edu.vn
14. Dr Vui Thi Le, Hanoi University of Public Health, Vietnam ltv@huph.edu.vn
15. Dr Joseph Hicks, University of Leeds, UK J.P.Hicks@leeds.ac.uk
16. Mrs Judy Wright, University of Leeds, UK J.M.Wright@leeds.ac.uk
17. Dr Sumit Kane, University of Melbourne, Australia sumit.kane@unimelb.edu.au

*Corresponding author: London School of Hygiene and Tropical Medicine, 15-17 Tavistock place London WC1H 9SH. tolib.mirzoev@lshtm.ac.uk

Keywords:

Responsiveness, health systems, health services, Vietnam, South East Asia, Ghana, sub-Saharan Africa, mental health, realist synthesis, pregnancy

For peer review only - http://bmjopen.bmj.com/site/about/guidelines.xhtml

Abstract

Introduction

Health systems responsiveness is a key objective of any health system, yet it is the least studied of all objectives particularly in low- and middle-income countries. Research on health systems responsiveness highlights its multiple elements, for example dignity and confidentiality. Little is known, however, about underlying theories of health systems responsiveness, and the mechanisms through which responsiveness works. This realist synthesis contributes to bridging these two knowledge gaps.

Methods and analysis

In this realist synthesis we will utilise a four-step process, comprising: mapping of theoretical bases, formulation of programme theories, theory refinement, and testing of programme theories using literature and empirical data from Ghana and Vietnam. We will include theoretical and conceptual pieces, reviews, empirical studies and grey literature, alongside the primary data. We will explore responsiveness as entailing external and internal interactions within health systems. The search strategy will be purposive and iterative, with continuous screening and refinement of theories. Data extraction will be combined with quality appraisal, using appropriate tools. Each fragment of evidence will be appraised as it is being extracted, for its relevance to the emerging programme theories and methodological rigour. The extracted data pertaining to contexts, mechanisms and outcomes will be synthesised to identify patterns and contradictions. Results will be reported using narrative explanations, following established guidance on realist syntheses.

Ethics and dissemination

Ethics approvals for the wider RESPONSE study, of which this review is one part, were obtained from the ethics committees of the following institutions: London School of Hygiene and Tropical Medicine (ref: 22981), University of Leeds, School of Medicine (ref: MREC19-051), Ghana Health Service (ref: GHS-ERC 012/03/20) and Hanoi University of Public Health (ref: 020-149/DD-YTCC).

We will disseminate results through academic papers, conference presentations and stakeholder workshops in Ghana and Vietnam.

Registration details

Prospero registration: CRD42020200353. Full record: https://www.crd.york.ac.uk/prospero/display_record.php?ID=CRD42020200353

Article summary

Strengths and limitations of this study

- The review will identify, and bridge, the knowledge gaps in the peer-reviewed and grey literature on (a) theoretical underpinnings of health systems responsiveness and (b) the mechanisms through which responsiveness works within the contexts of low- and middle-income countries

For peer review only - http://bmjopen.bmj.com/site/about/guidelines.xhtml

For peer review only

- The iterative nature of searches, screening, data extraction and quality assessments is a key strength of our approach, which allows deep engagements with the literature across multiple disciplines and settings
- However, a potentially large scope of literature on various elements of health systems responsiveness would require careful management of the research team workload, through prioritisation of programme theories in the searches and the analysis
- The composition of a large multi-country team will enable drawing on different context-specific understandings of health systems responsiveness
- However, the large cross-country review team will require careful coordination, to ensure representation of different perspectives in the analysis and shared intellectual leadership and conclusions.

For peer review only

For peer review only - http://bmjopen.bmj.com/site/about/guidelines.xhtml

Introduction

Health systems responsiveness is a key goal of any national health system and is "...*when institutions... are cognisant and respond appropriately to the universally legitimate expectations of individuals... safeguarding of rights of patients to adequate... care*" [1 p.3]. . People, especially the most vulnerable, are more likely to use services if health systems are responsive to their expectations[2-4]. For example, evidence shows that people's trust in their health systems and feeling secure within health facilities can improve the uptake of maternal health services amongst pregnant women in Nigeria[5 6]. Similarly, issues of trust determine utilisation of maternal healthcare by women from ethnic minorities in remote areas of Vietnam[7 8]. Thus, responsive health systems can facilitate improvements in uptake of healthcare services and ensure adherence to treatment, and ultimately contribute towards enhanced patient welfare and equitable improvements in population health[3 9].

When considering all health systems goals (which include improved health, fair financial contribution and efficiency)[10 11], responsiveness is the least studied, particularly in low- and middle-income countries (LMICs)[12 13]. Research on measuring health systems responsiveness used a survey toolkit from the World Health Organisation, which highlights seven elements of responsiveness: dignity, autonomy, confidentiality, prompt attention, quality of amenities, access to support networks, and choice of service provider[1 2 4 14-17]. These elements stem from literature on quality of care and patient satisfaction[18]. The literature also highlights that interpretations of responsiveness are *context-sensitive* (e.g. expectations of dignity reflect political, democratic and policy environment[2]), and *vary across actors* (e.g. patients, and providers, reflecting their different powers[3 19]) and *health facilities* (e.g. public/private [2 3]). Responsiveness, therefore, is arguably a socially-constructed, rather than an 'absolute' and 'universally normative' concept. Recent work also emphasises that interactions between people and their health systems are central to understanding health systems responsiveness[12] and improving responsiveness should therefore address both the 'people' and the 'systems' sides of such interactions[12 20 21].

Little is known, however, about: (a) theoretical underpinnings of the current interpretation of health systems responsiveness and (b) underlying mechanisms through which health systems responsiveness works for different actors (such as communities, health workers, managers) and under which conditions. This realist synthesis (RS) will contribute to bridging these knowledge gaps through advancing theorisation of health system responsiveness and providing an in-depth understanding of key mechanisms of how responsiveness works for different health systems actors. We hope that results of this review will ultimately inform improvements to health systems responsiveness in Ghana, Vietnam and other LMICs.

This RS is being undertaken as part of the wider RESPONSE study, which is a mixed-methods realist evaluation of health systems responsiveness in Ghana and Vietnam. RESPONSE seeks to contribute to improving health systems responsiveness in LMICs through case studies of addressing neglected health needs of vulnerable groups in Ghana and Vietnam. As described in the RESPONSE protocol available elsewhere[22], in Ghana the study will be implemented in Greater Accra Region and in Vietnam we will work in Bắc Giang Province. The study is being implemented jointly by the London School of Hygiene and Tropical Medicine, University of Ghana, Ghana Health Service, Mental Health Authority of Ghana, Hanoi University of Public Health, University of Leeds and University of Melbourne.

For peer review only - http://bmjopen.bmj.com/site/about/guidelines.xhtml

Aim and questions

This realist synthesis will deepen the understanding of theoretical foundations of health systems responsiveness and the mechanisms through which health systems responsiveness works (or not) for different health systems actors in different contexts. Through developing, testing and validating programme theories we will identify and explain which contexts trigger specific mechanisms through which health systems responsiveness can produce the intended or unintended outcomes. In the process, we will also highlight any gaps in the existing literature following our review. This RS will answer the following questions:

1. Which substantive theories underpin the understanding of health systems responsiveness in the literature, and how do they inform these interpretations?
2. In what way does health systems responsiveness work for different health systems actors (service users, providers and managers) in the contexts of low and middle-income countries?

The main result from this review will be detailed narrative explanations[23] of how health systems responsiveness works for different health systems actors in LMICs. These explanations will draw upon available theoretical and empirical literature, and insights from fieldwork in Ghana and Vietnam.

Methods and analysis

This RS uses a realist approach, which helps understand complex programmes by identifying how the multiple components interact in non-linear ways[24 25]. It is guided by an overall question of 'what works for whom, under which circumstances and why'[25], and is particularly appropriate for exploring the socially-constructed phenomena. A realist approach recognises micro (individual), meso (organisational) and macro (systemic) contexts (Cs) in triggering the mechanisms, which comprise reasoning and resources (Ms), to produce intended or unintended outcomes (Os) – altogether known as CMO configurations[26-31]. Such an approach is particularly suited to understanding the complexity of health systems responsiveness in diverse settings.

Realist synthesis, which applies realist logic to a systematic review methodology, provides in-depth understanding of complex phenomena through articulating theories shaped as CMO configurations that explain why, when and for whom the programmes or interventions work[23 24]. However, in contrast with traditional systematic reviews, realist reviews are *"…not a method or formula, but a logic of enquiry that is inherently pluralist and flexible, embracing both qualitative and quantitative, formative and summative, prospective and retrospective…"*[32 p.32]. Furthermore, RSs are more inclusive of the types of studies that can be included[23] and often incorporate more substantial consideration of grey literature[32].

Conceptually, RS involves broad phases which mirror the realist evaluation process: formulating questions, developing initial theories, testing and refining theories[33], all culminating in the final narrative[23]. Specific steps in conducting RS are close to those of the systematic reviews: identifying review questions, searching for primary studies, quality assessment, data extraction, synthesising results and dissemination[23 24]. However, these steps are not linear and, in contrast with traditional systematic reviews, overlap and are highly iterative.

Another key distinctive feature of the RS approach is that further to engagements with the literature, researchers also engage with key stakeholders, for example, in formulating initial theories

For peer review only - http://bmjopen.bmj.com/site/about/guidelines.xhtml

and developing policy recommendations[32]. However, there is no clear guidance on involvement of stakeholders in realist syntheses[34] with the RAMESES standards for realist syntheses being intentionally flexible and just referring to achievement of end-user relevance[35]. As a result, there is a wide variation in the degrees to which stakeholder engagements are approached in RSs, which include: informal consultations in formulating initial theories and developing policy recommendations[32] and formal data collection and analysis for example using in-depth interviews and surveys, to inform theory refinement[36] and even theory testing[33].

In this RS, we are guided by the RAMESES publications standards for realist syntheses[35] (see supplementary file) and will follow a four-step process similar to Cooper et al[33], which will be embedded throughout the RESPONSE study (Figure 1).

Step 1 will involve mapping of theoretical underpinnings of health systems responsiveness in low- and middle-income countries[23 33], through initial screening of literature. During this step, we will identify and analyse exclusively theoretical literature, which can help understand, and explain, health systems responsiveness. The main output from this step will be advanced theorisation of health systems responsiveness.

During *step 2,* we will formulate initial theories of how health systems responsiveness works. We will draw on theorisation of responsiveness from the previous step, team discussions and initial consultations with key health systems actors[32 33] in Ghana and Vietnam. During this step, we will potentially widen the scope of the literature review to include empirical studies on health systems responsiveness and will also review policy documentation in Ghana and Vietnam. Initial stakeholder consultations will be in the form of in-depth interviews with purposefully-identified key health systems actors (service users, communities, service providers and managers). These interviews are planned during the baseline data collection within RESPONSE to understand what responsiveness means to different health systems actors including its importance, underlying principles, components, mechanisms and intended outcomes[22], and analysis of interview data will contribute to formulation of initial programme theories. The main output from this step will be a (longer) list of programme theories of how health systems responsiveness works in LMICs.

In *step 3*, we will refine programme theories through continuing the purposeful screening of the literature alongside iterative engagements with key actors[32] in Ghana and Vietnam. Our engagements with key actors will utilise the planned intervention co-production workshops. In each country, we will aim to organise 4-6 intervention co-production workshops involving key actors (communities, service providers, facility managers, regional/province and national-level actors). These workshops will be led by relevant health authorities and facilitated by researchers. We will carefully document stakeholder views of causal pathways of how the interventions are intended to work to inform theory refinement, and through participant observations will also reflect on the co-production processes[22]. The interventions to improve health systems responsiveness will not be targeting individual elements of responsiveness in the WHO framework[1] but will seek to improve internal interactions (i.e. within health system) and external interaction (i.e. people-systems), through series of participatory workshops with health workers and communities[12 22]. The main output from this step will be a (shorter) list of selected programme theories for subsequent testing.

Finally, *step 4* will entail testing of programme theories. This will be done through using insights from empirical evaluations of implemented interventions in Ghana and Vietnam and through analysing empirical literature[33]. During this step, we will review literature that supports or refutes the programme theories and will use analytical insights from empirical evaluations of the interventions.

For peer review only - http://bmjopen.bmj.com/site/about/guidelines.xhtml

We will draw upon an approach used by Cooper et al where interview data were integrated with evidence from the literature in testing programme theories[33]. We already plan to evaluate effectiveness of interventions within the RESPONSE[22], and we will use this primary dataset for testing our programme theories. In each country, data collection will include combinations of about 60 in-depth interviews and 4-6 focus groups with purposefully identified key health systems actors (communities and service users, service providers), a multi-stage clustered household community survey with sample size estimated at 562 and review of relevant policy and facility documentation. Data collection tools, such as question guides for in-depth interviews and focus groups and survey questionnaire, will be structured by the selected programme theories and the empirical data will analysed retroductively (i.e. both inductively and deductively[13 22 37] as described in the data analysis later) alongside the insights from the literature, to test programme theories. The main output from this step will comprise results synthesised into narrative explanations[23] of how health systems responsiveness works for different health systems actors in LMICs.

Eligibility

In line with established principles on eligibility of evidence for RSs[23 32], we will not impose any strict restrictions on the study types and will include all available studies which focus on responsiveness of health and other public systems within LMICs. Specifically, we will include: (a) theoretical and conceptual pieces, opinions and analyses; (b) reviews: systematic reviews, scoping reviews, meta-syntheses, realist syntheses; (c) empirical studies: including qualitative studies, policy studies, randomised controlled trials (RCTs), quasi-experimental studies and cross sectional and cohort studies; and (d) grey literature, including discussion pieces, reports, policies, plans and guidelines in Ghana and Vietnam and relevant local-level documentation within health facilities.

However, we will exclude: (a) empirical (but not theoretical) studies published before 2000 i.e. the year when the WHO introduced health systems responsiveness as an explicit health systems objective; (b) studies with no full texts available for subsequent analysis; and (c) studies in languages where we are unable to source translation. The review is being conducted by a team comprising speakers of English, Spanish, French, Russian and Vietnamese. While initial screening will be conducted in either of these languages, analysis will be conducted in English and we will aim to either obtain an English copy or arrange for a translation where possible.

Next, we summarise eligibility following a participants-interventions-comparison/control-outcomes (PICO) approach.

Health systems responsiveness affects a range of different populations such as communities, individuals, service providers, facility managers, regional or provincial and national-level policymakers[1 12 18]. Therefore, will be no restriction on population groups. Evidence suggests that vulnerable individuals are especially dependant on the degree of health systems responsiveness to their needs[38 39]. In this review, while we will not exclude any populations, we will pay particular attention to responsiveness to pregnant women suffering from mental health conditions[22]. We anticipate this group will provide a depth of insight into responsiveness as mental health is typically the most neglected aspect of maternal health[40 41] and that responsive health systems should recognise such intersectional nature of vulnerability.

Our overall ‘intervention’ for improved health systems responsiveness will be two types of interactions which underpin the RESPONSE’s theoretical framework[22]: internal interactions (i.e.

For peer review only - http://bmjopen.bmj.com/site/about/guidelines.xhtml

within health systems such as between service providers and managers) and external interactions (i.e. between service users and health systems, typically at the point of service provision)[12].

Health systems responsiveness is a complex intervention, therefore the control is not applicable to this review. However, we will compare the CMO configurations across the different LMIC settings where it is feasible.

Our overarching outcome is improved health systems responsiveness, reflected in improved interactions, which follow the socially-constructed conceptualisation of responsiveness within RESPONSE[12 20-22].

Instead of separately considering each of seven elements of health systems responsiveness from the WHO framework[1] as distinct outcomes, we will seek to understand improvement in responsiveness of health systems in LMICs as a whole. In the process, some of the WHO elements of responsiveness may be aggregated into fewer and broader categories. As the review progresses, further outcomes may also emerge within specific CMO configurations.

Literature Searches

Searching for theoretical and empirical evidence for realist reviews are highly iterative. Multiple searches will be conducted using a variety of appropriate search techniques[42]. Conventional database searches will be complemented with CLUSTER searches techniques such as citation snowballing and project searching (e.g. WHO responsiveness tool project)[43]. These techniques will be used to identify related relevant studies and 'contextually rich' evidence.

Conceptually, RS involves four different types of searches at different stages of the review: a background search to get a feel of the literature, progressive searches to identify programme theories, an evidence search for evaluation studies to test chosen theories, and a final search once the review is almost complete to refine theories[32 42]. We will follow such an approach throughout our four steps, our search strategy will be purposive rather than exhaustive, and will evolve as our programme theories develop.

In answering the first review question (underlying theories of responsiveness), our searches will not be limited to any geographic area, whereas in answering the second review questions (how responsiveness works) will focus on the literature from LMICs. We will search combinations of:

1. Published literature from eight scientific databases: Applied Social Science Index Abstracts, CINAHL, Global Health, Maternity and Infant Care Database, Medline, PsycINFO, Scopus, Web of Science.
2. Grey literature from main gateways (e.g. ELDIS) and relevant organisations (e.g. World Health Organisation repository, MeasureEvaluation).
3. Health systems policy and regulatory documentation from Ghana and Vietnam.

At step 1, we will search Medline (1946+) and Google Scholar for reports containing substantive theories and frameworks. MeSHs, free text words and synonyms will be used for the following search concepts (see supplementary file for a sample Medline search strategy):

- *health systems responsiveness*: responsiveness/satisfaction/confidence/trust and WHO elements of responsiveness: dignity; autonomy; confidentiality; prompt attention; quality of amenities; access to support networks; and choice of service provider
- *theories*: theory; framework; conceptualisation

For peer review only - http://bmjopen.bmj.com/site/about/guidelines.xhtml

During step 2, we will widen the search from the previous step to also include empirical literature on health systems responsiveness within LMICs and systems policy and regulatory documentation from Ghana and Vietnam.

At steps 3 and 4, targeted searches for empirical and theoretical literature on how health systems responsiveness works in LMICs will be determined by the selected programme theories. MeSHs, free text words and synonyms will be used for the following search concepts:

- *different health systems actors*: communities/pregnant women/service providers/doctors/nurses/health workers/ health managers/health providers/policymakers/decision-makers/implementers/researchers/
- *LMIC* contexts, specifically sub-Saharan Africa and South East Asia
- *programme theory* being tested.

Search results will be managed using EndNote version X9 software. Records will be uploaded into Rayyan software (https://rayyan.qcri.org/welcome) for initial screening as appropriate. Qualitative data analysis software NVIVO will be used for organising and managing data, cross referencing concepts, actors and populations. Data extraction will be done with Microsoft Word or Excel.

Screening in RSs is typically done throughout the review as the searches progress, rather than in one large exercise as in the systematic reviews[23]. In our review, we will conduct: (a) initial screening for theoretical frameworks and models of health systems responsiveness during step 1 and then (b) purposeful screening in steps 2-4 for developing, refining and testing programme theories. Screening in each step will be done collaboratively by the different team members, and we will ensure that a minimum of 20% of results are double-screened for calibration and quality control.

Data extraction

Realist syntheses have no standardised common data extraction form and data extraction is often combined with quality appraisal of studies[23 24 33]. As RS involves a highly iterative process with search terms continually refined, the process will be documented carefully for reporting.

During all steps, a data extraction table in Microsoft Word or Excel format will be used to track included papers, grey literature and policy documents, adapting the following headings to each step of the RS:

a) key paper identifiers (full citation)
b) type of paper (published, grey literature)
c) social science theory introduced and/or used
d) elements of health systems responsiveness addressed[1 12]
e) links to selected programme theory(ies) showing causality in CMO configurations, through identifying evidence on which key contexts trigger specific mechanisms to produce intended or unintended outcomes.

In step 1 of our RS, we will specifically focus on understanding theorisation of responsiveness and will utilise and potentially expand headings a-c. In steps 2-4, we will add headings d-e as applicable to stages of theory development, refinement and testing.

For peer review only - http://bmjopen.bmj.com/site/about/guidelines.xhtml

The primary data collection in step 4 will involve tailored topic guides for interviews and focus groups, which will be structured around the selected programme theories. Transcripts from the interviews and focus groups will be coded by the CMO configurations (or their fragments) to help refine, discard or consolidate the causality within selected programme theories during the analysis.

Quality assessment

Quality assessment, data extraction and collation of stakeholder opinions are typically combined in RS[32], a process which we will also follow in our review.

All studies will be considered for their *relevance* to programme theories of health systems responsiveness, specifically ability to provide insights into contexts, mechanisms and outcomes and *rigour*, commensurate to respective study types[23 32].

Quality appraisal in RS is usually done on case-by-case basis and we will adapt and utilise appropriate tools (e.g. JBI or CASP quality appraisal tools[44]) and use applicable guidance (e.g. RAMESES standards[35 45]). Assessing relevance and rigour will be undertaken collaboratively through regular team discussions. Quality assessment will be conducted on each fragment of evidence as it is being extracted, rather on whole studies.

Data analysis and synthesis

We will undertake a retroductive approach to analysis meaning, combining both inductive and deductive reasoning logics together[13 22 37]. Such an approach is a distinctive feature of realist studies, which also draws upon 'insights or hunches' to help identify the hidden causal forces behind the identified patterns such as outcomes[37].

Analysis of sub-groups in RS normally emerges as part of the theory elicitation process[24 32]. Therefore, it would be inappropriate to narrowly pre-determine the subgroups. The literature on RS also distinguishes specific and iterative steps in the data synthesis, for example organising extracted data into evidence tables, theming by individual reviewers, formulating chains of inference (CMO connections at theory and sub-theory levels) from the themes, linking chains of inference and hypothesis formulation[23].

Analysis of theorisation of health systems responsiveness in step 1 will not be restricted to any geographical, health condition or population sub-sets. As the review develops, from step 2 onwards we will add focus on empirical literature from the LMICs. We are particularly interested in maternal mental health as a possible condition sub-set, and key health systems actors (service users, service providers and managers) as a population sub-set. However, the subgroup analysis will be fully driven by the theory elicitation.

We will relate results of our analyses within these subsets to the health systems responsiveness as a whole, reflected in internal and external interactions within health systems. Extracted data pertaining to contexts, mechanisms and outcomes will be synthesised to identify patterns and contradictions in the relationships between contexts, mechanisms and outcomes. These relationships will be documented and will inform initial theories from step 2, to be subsequently refined and tested during steps 3 and 4, respectively. Data synthesis will also lead us to identify

For peer review only - http://bmjopen.bmj.com/site/about/guidelines.xhtml

social science theories that may further explain our programme theories. At this stage, middle-range theories will be identified and extracted.

Our synthesis will be specifically concerned with understanding the conditions under which health systems responsiveness works (or not) for different health systems actors. Data synthesis in RS takes several forms, essentially entailing a form of 'triangulation', bringing together information from different studies to explain why a pattern of outcomes may occur[32 33]. Our review would go further and would identify explanations for potentially contrasting (or similar) findings to identify the circumstances in which the intended mechanisms and intended outcomes occur (improved health systems responsiveness, reflected in improved interactions) and those in which unintended mechanisms and outcomes (e.g. sense of poor interactions due to mutual distrust) occur.

We will specifically synthesise data along our conceptualisations of health systems responsiveness (responsiveness as a whole, internal and external interactions[12] and possibly relevant elements where applicable[1]). These will relate closely to our programme theories and further elements of data synthesis may be added to capture the causality and contingent nature of the CMO configurations. In reporting results, we will produce rich narrative explanations of our programme theories[23], following established guidance on reporting realist syntheses[23 24 35].

Patient and public involvement

This review will use published data alongside primary data. We do not envisage any patient and public involvement during step 1 (theorisation of health systems responsiveness). However, patients and members of the public will be involved in steps 2-4 as part of the wider stakeholder engagement. In step 2, their involvement would be as sources of local knowledge in identifying priority areas for responsiveness during initial consultations. In step 3, their involvement would comprise more active participation in the iterative engagements during intervention co-production. In step 4, patient and public involvement would be evaluative of the effects of interventions.

Ethics and dissemination

Ethics approvals for the wider RESPONSE study, which also cover the primary data collection for the realist synthesis, were obtained from the London School of Hygiene and Tropical Medicine (ref: 22981), the University of Leeds School of Medicine (ref: MREC19-051), Ghana Health Service (ref GHS-ERC 012/03/20) and Hanoi University of Public Health (ref 020-149/DD-YTCC).

We will disseminate results through academic papers and stakeholder workshops in Ghana and Vietnam. The findings will also be presented at national and international scientific conferences, such as the biannual Global Symposia on Health Systems Research.

Figure 1: Realist synthesis within the RESPONSE study

For peer review only - http://bmjopen.bmj.com/site/about/guidelines.xhtml

References

1. de Silva A. A framework for measuring responsiveness. GPE Discussion Paper series No 32. Geneva: World Health Organization, 2000.
2. Robone S, Rice N, Smith PC. Health Systems' Responsiveness and Its Characteristics: A Cross-Country Comparative Analysis. *Health Serv Res* 2011;46(6pt2):2079-100. doi: 10.1111/j.1475-6773.2011.01291.x
3. Valentine N, Prasad A, Rice N, et al. Health Systems Responsiveness – A Measure of the Acceptability of Health Care Processes and Systems. In: Smith P, Mossialos E, S L, eds. Performance Measurement for Health System Improvement: Experiences, Challenges and Prospects. London: WHO European Regional Office 2009:pp. 138–86.
4. Darby C, Valentine N, Murray CJL, et al. World Health Organization: Strategy on measuring responsiveness. *The Journal of Medicine and Philosophy* 2000;39(1):2.
5. Etiaba E, Manzano A, Agbawodikeizu U, et al. "If you are on duty, you may be afraid to come out to attend to a person": fear of crime and security challenges in maternal acute care in Nigeria from a realist perspective. *BMC Health Services Research* 2020;20(1):903. doi: 10.1186/s12913-020-05747-9
6. Mirzoev T, Etiaba E, Ebenso B, et al. Tracing theories in realist evaluations of large-scale health programmes in low- and middle-income countries: experience from Nigeria. *Health Policy Plan* 2020;35(9):1244-53. doi: 10.1093/heapol/czaa076
7. Doan DTT, Bui HTT, Le TM, et al. Utilization of services provided by village based ethnic minority midwives in mountainous villages of Vietnam. *International Journal of Women's Health* 2016;8:571-80. doi: 10.2147/IJWH.S112996
8. Doan DTT, Mirzoev T, Nguyen CC, et al. Utilization of Services Provided by Village-Based Ethnic Minority Midwives in Vietnam: Lessons From Implementation Research. *J Public Health Manag Pract* 2018;24:S9-S18. doi: 10.1097/phh.0000000000000689
9. Lodenstein E, Dieleman M, Gerretsen B, et al. A realist synthesis of the effect of social accountability interventions on health service providers' and policymakers' responsiveness. *Systematic Reviews* 2013;2(1):98.
10. WHO. Everybody business : strengthening health systems to improve health outcomes : WHO's framework for action. Geneva: World Health Organization 2007.
11. WHO. The World Health Report 2000. Health Systems: Improving Performance. Geneva: World Health Organization 2000.
12. Mirzoev T, Kane S. What is health systems responsiveness? Review of existing knowledge and proposed conceptual framework. *BMJ Global Health* 2017;2(4):e000486. doi: 10.1136/bmjgh-2017-000486
13. Lodenstein E, Dieleman M, Gerretsen B, et al. Health provider responsiveness to social accountability initiatives in low- and middle-income countries: a realist review. *Health Policy Plan* 2016;32(1):125-40. doi: 10.1093/heapol/czw089
14. Letkovicova H, Prasad A, Vallée RL, et al. The health systems responsiveness analytical guidelines for surveys in the multi-country survey study. Geneva: World Health Organization 2005.
15. Chao J, Lu B, Zhang H, et al. Healthcare system responsiveness in Jiangsu Province, China. *BMC Health Services Research* 2017;17(1):31. doi: 10.1186/s12913-017-1980-2
16. Forouzan S, Padyab M, Rafiey H, et al. Measuring the Mental Health-Care System Responsiveness: Results of an Outpatient Survey in Tehran. *Frontiers in Public Health* 2015;3:285. doi: 10.3389/fpubh.2015.00285
17. Malhotra C, Do YK. Public health expenditure and health system responsiveness for low-income individuals: results from 63 countries. *Health Policy Plan* 2017;32(3):314-19. doi: 10.1093/heapol/czw127 [published Online First: 2016/09/22]
18. Valentine N, de Silva A, Kawabata K, et al. Chapter 43: Health System Responsiveness: Concepts, Domains and Operationalization. In: Murray C, Evans D, eds. Health systems performance

For peer review only - http://bmjopen.bmj.com/site/about/guidelines.xhtml

assessment : debates, methods and empiricism. Geneva: World Health Organization 2003:pp 573-96.
19. Valentine NB, Bonsel GJ. Exploring models for the roles of health systems' responsiveness and social determinants in explaining universal health coverage and health outcomes. *Global Health Action* 2016;9:10.3402/gha.v9.29329. doi: 10.3402/gha.v9.29329
20. Cleary S, Molyneux S, Gilson L. Resources, attitudes and culture: an understanding of the factors that influence the functioning of accountability mechanisms in primary health care settings. *BMC Health Services Research* 2013;13(1):320.
21. Berlan D, Shiffman J. Holding health providers in developing countries accountable to consumers: a synthesis of relevant scholarship. *Health Policy Plan* 2012;27(4):271-80. doi: 10.1093/heapol/czr036
22. Mirzoev T, Manzano A, Ha BTT, et al. Realist evaluation to improve health systems responsiveness to neglected health needs of vulnerable groups in Ghana and Vietnam: Study protocol. *PLoS One* 2021;16(1):e0245755. doi: 10.1371/journal.pone.0245755
23. Rycroft-Malone J, McCormack B, Hutchinson AM, et al. Realist synthesis: illustrating the method for implementation research. *Implementation Science* 2012;7(1):33. doi: 10.1186/1748-5908-7-33
24. Pawson R, Greenhalgh T, Harvey G, et al. Realist synthesis: an introduction. ESRC Research Methods Programme, Working Paper Series. 2004.
25. Pawson R, Tilley N. Realistic Evaluation: London: SAGE Publications 1997.
26. Greenhalgh T, Pawson R, Wong G, et al. 'Theory' in realist evaluation: The Rameses II project 2017.
27. Greenhalgh T, Pawson R, Wong G, et al. What is a mechanism? What is a programme mechanism?: The Rameses II project 2017.
28. Greenhalgh T, Pawson R, Wong G, et al. What realists mean by context; or, Why nothing works everywhere or for everyone: The Rameses II project 2017.
29. Manzano A. The craft of interviewing in realist evaluation. *Evaluation* 2016;22(3):342–60. doi: 10.1177_1356389016638615
30. Mirzoev T, Etiaba E, Ebenso B, et al. Study protocol: realist evaluation of effectiveness and sustainability of a community health workers programme in improving maternal and child health in Nigeria. *Implementation Science* 2016;11(1):1-11. doi: 10.1186/s13012-016-0443-1
31. Pawson R, Manzano-Santaella A. A realist diagnostic workshop. *Evaluation* 2012;18(2):176-91. doi: 10.1177/1356389012440912
32. Pawson R, Greenhalgh T, Harvey G, et al. Realist review--a new method of systematic review designed for complex policy interventions. *J Health Serv Res Policy* 2005;10 Suppl 1:21-34. doi: 10.1258/1355819054308530 [published Online First: 2005/08/02]
33. Cooper C, Lhussier M, Shucksmith J, et al. Protocol for a realist review of complex interventions to prevent adolescents from engaging in multiple risk behaviours. *BMJ Open* 2017;7(9):e015477. doi: 10.1136/bmjopen-2016-015477
34. Abrams R, Park S, Wong G, et al. Lost in reviews: Looking for the involvement of stakeholders, patients, public and other non-researcher contributors in realist reviews. *Research Synthesis Methods* 2021;12(2):239-47. doi: https://doi.org/10.1002/jrsm.1459
35. Wong G, Greenhalgh T, Westhorp G, et al. RAMESES publication standards: realist syntheses. *BMC Medicine* 2013;11(1):21. doi: 10.1186/1741-7015-11-21
36. Dunn J, Martin RA, Hackney JJ, et al. Early vocational rehabilitation for people with spinal cord injury: a research protocol using realist synthesis and interviews to understand how and why it works. *BMJ Open* 2021;11(5):e048753. doi: 10.1136/bmjopen-2021-048753
37. Greenhalgh T, Pawson R, Wong G, et al. Retroduction in realist evaluation: The Rameses II project 2017.

For peer review only - http://bmjopen.bmj.com/site/about/guidelines.xhtml

38. Klode K, Ringer A, Hølge-Hazelton B. Interprofessional and intersectoral collaboration in the care of vulnerable pregnant women: An interpretive study. *Journal of Interprofessional Care* 2020:1-10. doi: 10.1080/13561820.2020.1761306
39. UNDP. Human Development Report 2016 Human Development for Everyone: UNDP 2016.
40. Cheung FK, Snowden LR. Community mental health and ethnic minority populations. *Community Ment Health J* 1990;26(3):277-91. doi: 10.1007/bf00752778
41. Bagadia A, Chandra PS. Starting the conversation - Integrating mental health into maternal health care in India. *The Indian journal of medical research* 2017;145(3):267-69. doi: 10.4103/ijmr.IJMR_910_16
42. Booth A, Briscoe S, Wright JM. The "realist search": A systematic scoping review of current practice and reporting. *Research Synthesis Methods* 2020;11(1):14-35. doi: https://doi.org/10.1002/jrsm.1386
43. Booth A, Harris J, Croot E, et al. Towards a methodology for cluster searching to provide conceptual and contextual "richness" for systematic reviews of complex interventions: case study (CLUSTER). *BMC Medical Research Methodology* 2013;13(1):118. doi: 10.1186/1471-2288-13-118
44. Hannes K, Lockwood C, Pearson A. A Comparative Analysis of Three Online Appraisal Instruments' Ability to Assess Validity in Qualitative Research. *Qual Health Res* 2010;20(12):1736-43. doi: 10.1177/1049732310378656
45. Wong G, Westhorp G, Manzano A, et al. RAMESES II reporting standards for realist evaluations. *BMC Medicine* 2016;14(1):96. doi: 10.1186/s12916-016-0643-1

Authors' contributions

TM and SK conceived the study; TM, AC, AM, IA, MEA, ADA, LG, LY, EA, BTTH, DTTT, QCTN, LMT, LVT, JH, JW, SK substantially contributed to the design of the work, jointly wrote the protocol, read and approved the final version of the manuscript.

Funding statement

The research protocol reported in this paper received funding from the Joint MRC/ESRC/DFID/Wellcome Health Systems Research Initiative (grant ref: MR/T023481/1). The views expressed in this publication are those of the author(s) and not necessarily those of the funders.

Competing interests statement

None declared

Word count

3,812 words

For peer review only - http://bmjopen.bmj.com/site/about/guidelines.xhtml

For peer review only

For peer review only - http://bmjopen.bmj.com/site/about/guidelines.xhtml

Figure 1: Realist synthesis within the RESPONSE study

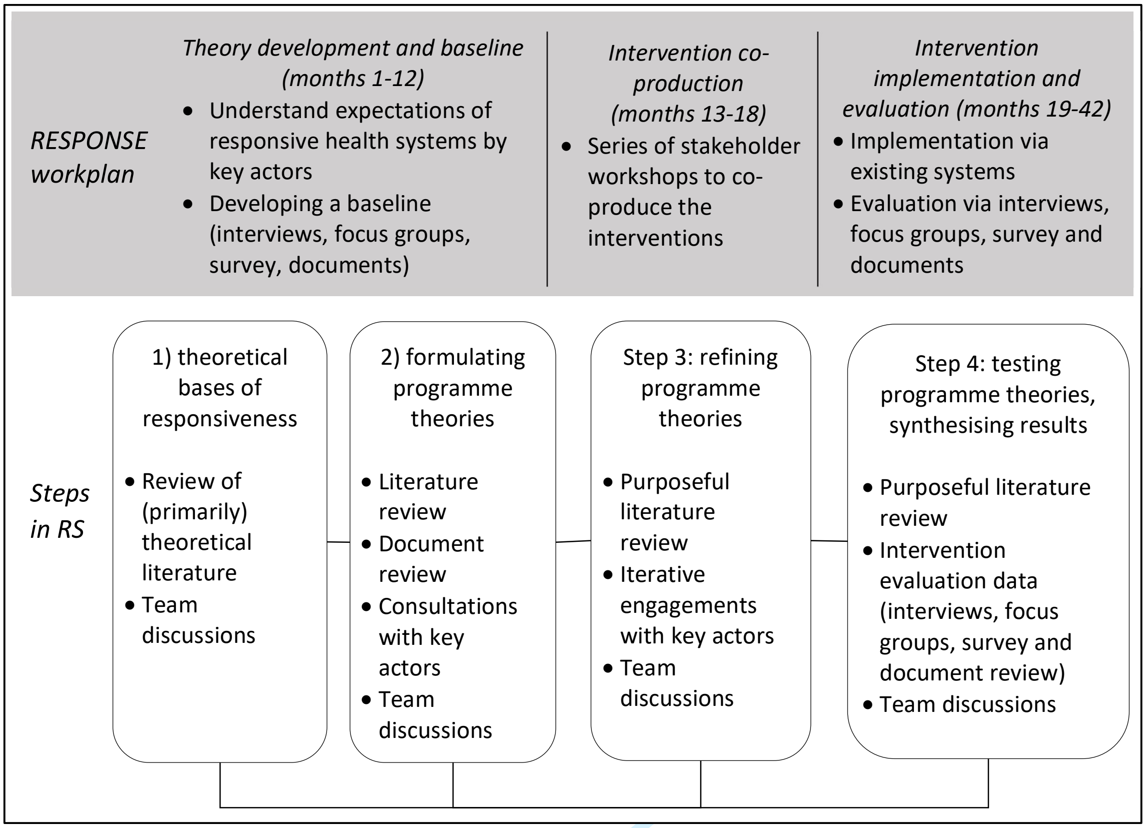

For peer review only - http://bmjopen.bmj.com/site/about/guidelines.xhtml

Literature search for theories of Health Service Responsiveness

Ovid MEDLINE(R) and Epub Ahead of Print, In-Process & Other Non-Indexed Citations and Daily <1946 to November 05, 2020>

Search date: 06-11-2020

Search Strategy:

----

1 conceptual*.tw. (92761)

2 theor*.tw. (656075)

3 ((framework or concep* or logic) adj2 (model* or analy* or evaluat*)).tw. (39784)

4 (reason? adj3 (non-responsiv* or responsiv*)).tw,kw. (42)

5 (factor? adj3 (non-responsiv* or responsiv*)).tw,kw. (4832)

6 (determin* adj3 (non-responsiv* or responsiv*)).tw,kw. (2120)

7 ((concept* or framework or logic or model*) adj3 (non-responsiveness or responsiv*)).tw,kw. (1113)

8 Comment/ (876294)

9 Letter/ (1106450)

10 Editorial/ (545898)

11 "Comment on".ti. (28606)

12 (letter* adj3 editor*).ti. (18269)

13 or/1-12 [Theory Search] (2660960)

14 (health* adj2 system* adj5 respons*).tw,kw. (1718)

15 (health* adj2 delivery* adj5 respons*).tw,kw. (197)

16 (health* adj2 delivery* adj5 (confiden* or trust*)).tw,kw. (78)

17 (health* adj2 system* adj5 (confiden* or trust*)).tw,kw. (640)

18 (health* adj2 system* ad5 satisf*).tw,kw. (0)

19 (health* adj2 delivery* adj5 satisf*).tw,kw. (147)

For peer review only

20 (health* adj2 system* adj5 (prompt or timely or lengthy or delay*)).tw,kw. (540)

21 (health* adj2 delivery* adj5 (prompt or timely or lengthy or delay*)).tw,kw. (61)

22 (health* adj2 system* adj5 dignity).tw,kw. (9)

23 (health* adj2 delivery* adj5 dignity).tw,kw. (0)

24 (health* adj2 system* adj5 (choice? or choose or decide? or decision*)).tw,kw. (1293)

25 (health* adj2 delivery* adj5 (choice? or choose or decide? or decision*)).tw,kw. (348)

26 (health* adj2 system* adj5 access*).tw,kw. (2430)

27 (health* adj2 delivery* adj5 access*).tw,kw. (326)

28 (health* adj2 system* adj5 quality).tw,kw. (2670)

29 (health* adj2 delivery* adj5 quality*).tw,kw. (1240)

30 (health* adj2 system* adj5 accountab*).tw,kw. (252)

31 (health* adj2 delivery* adj5 accountab*).tw,kw. (67)

32 who responsiv*.tw,kw. (11)

33 or/21-32 [Health Service reponsiveness Text word search] (11370)

34 exp *Patient Satisfaction/ (35253)

35 exp *Health Services Accessibility/ (60867)

36 *personal autonomy/ (6110)

37 *respect/ (193)

38 *trust/ (4075)

39 *Confidentiality/ (11479)

40 or/31-39 (116522)

41 exp *"delivery of health care, integrated"/ or *"delivery of health care"/ (69168)

42 40 and 41 [Healthcare Responsivity MeSH search] (3985)

43 33 or 42 [Healthcare Responsivity search] (15163)

52 13 and 43 [Theories of Healthcare Responsivity] (1802)

For peer review only

Manuscript Title**:** Protocol for a realist synthesis of health systems responsiveness in low- and middle-income countries.

Checklist for reporting realist evaluations. Source: Wong G, Greenhalgh T, Westhorp G, et al. RAMESES publication standards: realist syntheses. *BMC Medicine* 2013;11(1):21. doi: 10.1186/1741-7015-11-21 https://bmcmedicine.biomedcentral.com/articles/10.1186/1741-7015-11-21

**TITLE**			**Included Y/N**	**Page No**
1		In the title, identify the document as a realist synthesis or review	Y	1
ABSTRACT				
2		While acknowledging publication requirements and house style, abstracts should ideally contain brief details of: the study's background, review question or objectives; search strategy; methods of selection, appraisal, analysis and synthesis of sources; main results; and implications for practice.	Y	2
INTRODUCTION				
3	Rationale for review	Explain why the review is needed and what it is likely to contribute to existing understanding of the topic area.	Y	4
4	Objectives and focus of review	State the objective(s) of the review and/or the review question(s). Define and provide a rationale for the focus of the review.	Y	4-5
METHODS				
5	Changes in the review process	Any changes made to the review process that was initially planned should be briefly described and justified.	n/a for protocols	n/a
6	Rationale for using realist synthesis	Explain why realist synthesis was considered the most appropriate method to use.	Y	5
7	Scoping the literature	Describe and justify the initial process of exploratory scoping of the literature.	Y	7-8
8	Searching processes	While considering specific requirements of the journal or other publication outlet, state and provide a rationale for how the iterative searching was done. Provide details on all the sources accessed for information in the review. Where searching in electronic databases has taken place, the details should include, for example, name of database, search terms, dates of coverage and	Y	7-8

For peer review only

TITLE			Included Y/N	Page No
		date last searched. If individuals familiar with the relevant literature and/or topic area were contacted, indicate how they were identified and selected.		
9	Selection and appraisal of documents	Explain how judgements were made about including and excluding data from documents, and justify these.	Y	6-7
10	Data extraction	Describe and explain which data or information were extracted from the included documents and justify this selection.	Y	9
11	Analysis and synthesis processes	Describe the analysis and synthesis processes in detail. This section should include information on the constructs analyzed and describe the analytic process.	Y	9-10
RESULTS				
12	Document flow diagram	Provide details on the number of documents assessed for eligibility and included in the review with reasons for exclusion at each stage as well as an indication of their source of origin (for example, from searching databases, reference lists and so on). You may consider using the example templates (which are likely to need modification to suit the data) that are provided.	n/a for protocols	n/a
13	Document characteristics	Provide information on the characteristics of the documents included in the review.	n/a for protocols	n/a
14	Main findings	Present the key findings with a specific focus on theory building and testing.	n/a for protocols	n/a
DISCUSSION				
15	Summary of findings	Summarize the main findings, taking into account the review's objective(s), research question(s), focus and intended audience(s).	n/a for protocols	n/a
16	Strengths, limitations and future research directions	Discuss both the strengths of the review and its limitations. These should include (but need not be restricted to) (a) consideration of all the steps in the review process and (b) comment on the overall strength of evidence supporting the explanatory insights which emerged.<br>The limitations identified may point to areas where further work is needed.	Y	2-3

For peer review only

TITLE			Included Y/N	Page No
17	Comparison with existing literature	Where applicable, compare and contrast the review's findings with the existing literature (for example, other reviews) on the same topic.	n/a for protocols	n/a
18	Conclusion and recommendations	List the main implications of the findings and place these in the context of other relevant literature. If appropriate, offer recommendations for policy and practice.	n/a for protocols	n/a
19	Funding	Provide details of funding source (if any) for the review, the role played by the funder (if any) and any conflicts of interests of the reviewers.	Y	13

For peer review only
